# Conserved and plant-unique strategies for overcoming endoplasmic reticulum stress

**DOI:** 10.3389/fpls.2014.00069

**Published:** 2014-02-26

**Authors:** Cristina Ruberti, Federica Brandizzi

**Affiliations:** ^1^Plant Research Laboratory, Department of Energy, Michigan State UniversityEast Lansing, MI, USA; ^2^Department of Plant Biology, Michigan State UniversityEast Lansing, MI, USA

**Keywords:** unfolded protein response, ER stress, UPR temporal activation, adaptation, autophagy, programed cell death, apoptosis, eukaryotes

## Abstract

Stress caused by environmental conditions or physiological growth can lead to an accumulation of unfolded proteins in the endoplasmic reticulum (ER) causing ER stress, which in turn triggers a cytoprotective mechanism termed the unfolded protein response (UPR). Under mild-short stress conditions the UPR can restore ER functioning and cell growth, such as reducing the load of unfolded proteins through the upregulation of genes involved in protein folding and in degrading mis-folded proteins, and through autophagy activation, but it can also lead to cell death under prolonged and severe stress conditions. A diversified suite of sensors has been evolved in the eukaryotic lineages to orchestrate the UPR most likely to suit the cell’s necessity to respond to the different kinds of stress in a conserved as well as species-specific manner. In plants three UPR sensors cooperate with non-identical signaling pathways: the protein kinase inositol-requiring enzyme (IRE1), the ER-membrane-associated transcription factor bZIP28, and the GTP-binding protein β1 (AGB1). In this mini-review, we show how plants differ from the better characterized metazoans and fungi, providing an overview of the signaling pathways of the UPR, and highlighting the overlapping and the peculiar roles of the different UPR branches in light of evolutionary divergences in eukaryotic kingdoms.

## INTRODUCTION

Environmental or physiological conditions that interfere with the proper protein folding in the endoplasmic reticulum (ER) lead to an accumulation of potentially toxic mis-folded proteins, a condition generally termed as “ER stress”. To restore ER homeostasis, a network of inter-organelle signaling pathways mediates the “unfolded protein response” (UPR), leading to an increase of protein folding capacity in the ER (Walter and Ron, 2011). If these mechanisms of adaptation and survival to ER stress fail, the UPR signaling leads the cells toward cell death (Hetz, 2012). Even if several aspects of the UPR are conserved across eukaryotes, the mechanisms to counteract the ER stress can vary across plants, metazoans and yeast (Kimata and Kohno, 2011; Chen and Brandizzi, 2013a; Howell, 2013). This review focuses on the current understanding of how the UPR signaling pathways initiate and progress in response to the severity and duration of ER stress and addresses the overlapping and unique roles of the UPR response in eukaryotes with emphasis on multicellular eukaryotes.

## UPR ARMS IN EUKARYOTES AND THEIR ER STRESS-SENSING MECHANISMS

**FIGURE 1:**
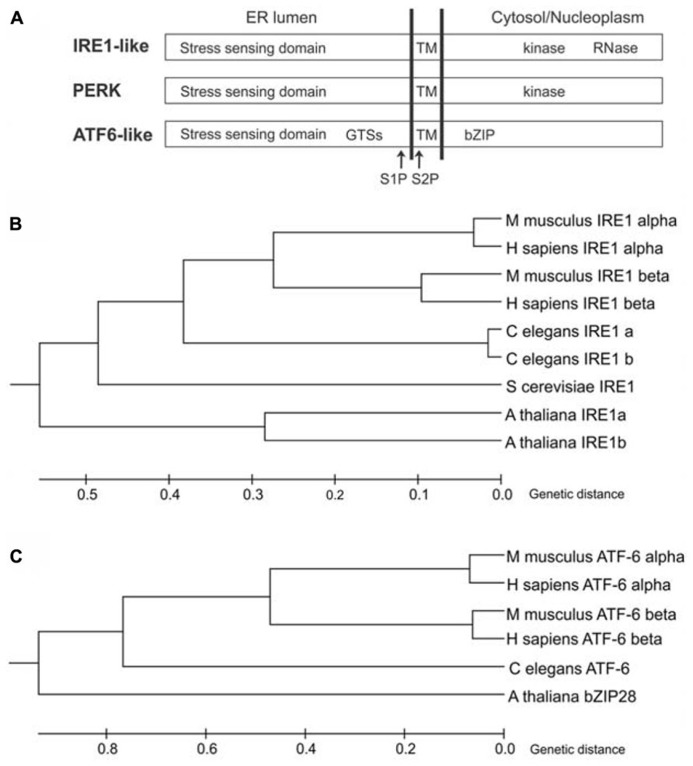
**ER stress-sensing proteins in eukaryotes. (A)** Schematic diagram of IRE1-like, PERK and ATF6-like proteins (not to scale). GTSs, Golgi trafficking signals; TM, transmembrane domain; S1P, site 1 protease cleavage site; S2P, site 2 protease cleavage site; bZIP, basic leucine zipper domain. **(B)** Phylogenetic tree analysis of IRE1-like proteins from *Animalia* (*Homo sapiens*, *Mus musculus* and *Caenorhabditis elegans*), *Fungi* (*Saccharomyces cerevisiae*) and *Plantae* (*Arabidopsis thaliana*) was constructed by the Unweighted Pair Group Method with Arithmetic Mean (UPGMA) method using MEGA6 software. NCBI reference sequences: IRE1 alpha (*H.s*)-NP_001424, IRE1 beta (*H.s*)-NP_150296, IRE1 alpha (*M.m*)-NP_076402, IRE1 beta (*M.m*)-NP_036146, IRE1a (*C.e*)-NP_001254135, IRE1b (*C.e*)-NP_001254136, IRE1 (*S.c*)-NP_011946, IRE1a (*A.t*)-NP_565419, IRE1b (*A.t*)-NP_568444. The tree is drawn to scale, with branch lengths in the same units as those of the evolutionary distances used to infer the phylogenetic tree. The evolutionary distances were computed using the Poisson correction method. **(C)** Phylogenetic tree analysis of ATF6-like proteins from *Animalia* (*H. sapiens*, *M. musculus* and *C. elegans*) and *Plantae* (*A. thaliana*) was constructed by the UPGMA method using MEGA6 software. NCBI reference sequences: ATF6 alpha (*H.s*)-NP_031374, ATF6 beta (*H.s*)-NP_001129625, ATF6 alpha (*M.m*)-NP_001074773, ATF6 beta (*M.m*)-NP_059102, ATF6 (*C.e*)-NP_510094, bZIP28 (*A.t*)-NP_187691. The tree is drawn to scale, with branch lengths in the same units as those of the evolutionary distances used to infer the phylogenetic tree. The evolutionary distances were computed using the Poisson correction method.

In the yeast *Saccharomyces cerevisiae*, the UPR is mediated by inositol-requiring enzyme1 (IRE1; Ire1p; Cox et al., 1993; Mori et al., 1993), an ER-resident protein largely conserved in eukaryotes. IRE1 is a type I transmembrane protein, with an N-terminal ER luminal stress-sensing domain, and a Ser/Thr kinase domain and an endoribonuclease domain in the cytosol (**Figure 1A**). Metazoans and plants have expanded their UPR signaling pathways with additional ER stress sensors (**Figures 1B,C**). Indeed, in metazoans, at least three ER transmembrane sensors initiate the UPR: IRE1 (IRE1α and IRE1β isoforms), the activating transcription factor 6 proteins (ATF6α and ATF6β isoforms), and the protein kinase RNA-like ER kinase (PERK) (Hetz, 2012). ATF6 is a type II transmembrane protein, characterized by a C-terminal ER lumen domain and an N-terminal cytosolic domain containing a bZIP (basic leucine zipper) transcriptional factor domain, while PERK is a type I transmembrane protein, with an ER-luminal stress-sensing domain and a cytosolic Ser/Thr kinase domain (**Figure 1A**). In plants, the UPR regulators so far identified are two IRE1 homologs (IRE1A and IRE1B; Koizumi et al., 2001), a functional homolog of ATF6 (bZIP28; Liu et al., 2007a), and a component of the G protein complex (AGB1; Wang et al., 2007). Intriguingly, the involvement of G protein complex in UPR has not been observed in other eukaryotes possibly because of a redundancy of the multiple isoforms of the heterotrimeric GTP-binding proteins in metazoans. Moreover, a functional PERK ortholog has not been identified in plants.

The mechanisms of how ER stress is sensed have been partially defined: the UPR sensors may detect ER stress (1) through the dissociation of their ER-luminal stress-sensing domain from the ER chaperones, which would be induced by the binding of ER chaperones to unfolded proteins, as shown for IRE1α, PERK, and ATF6 in metazoans (Kimata and Kohno, 2011); (2) through the direct binding of the ER-luminal domain of the UPR sensors to the unfolded proteins, as shown for IRE1 in yeast (Gardner and Walter, 2011); and (3) through post-translational modifications within the luminal domain, as observed in mammals for ATF6, where for example the hypoglycosylation is a monitoring mechanism for ER homeostasis to sense the glucose starvation or N-linked glycosylation impairment (Hong et al., 2004).

Upon ER stress induction, the UPR sensors are activated as follows: (1) the RNase domain of IRE1 is activated through its oligomerization and trans-autophosphorylation via its own kinase domain (Korennykh et al., 2009; Ali et al., 2011); (2) ATF6-orthologs are transported to the Golgi likely via specific trafficking signals (Shen et al., 2002; Srivastava et al., 2012) with the COPII vesicles (Schindler and Schekman, 2009; Srivastava et al., 2012), where the ATF6-orthologs are cleaved by the sequential action of the site 1 and site 2 proteases (S1P and S2P), thus releasing their N-terminal cytosolic transcription factor for translocation to the nucleus (Ye et al., 2000; Liu et al., 2007a); and (3) after PERK dimerization, one PERK homodimer likely inserts its flexible activation loop into the catalytic site of the adjacent homodimer, resulting in an interdimer trans-phosphorylation (Marciniak et al., 2006; Cui et al., 2011), that activates the kinase domain of PERKs. The mechanisms that lead to a role of AGB1 in the UPR are unknown (Wang et al., 2007; Chen and Brandizzi, 2012). In subcellular fractionation experiments, AGB1 has been found to be largely associated with the ER (Wang et al., 2007). Because AGB1 lacks a transmembrane domain, it is possible that post-translational modifications may modulate its UPR signaling function.

## THE ACTIVATION OF THE UPR ARMS: THE TRANSLATIONAL ATTENUATION

**FIGURE 2:**
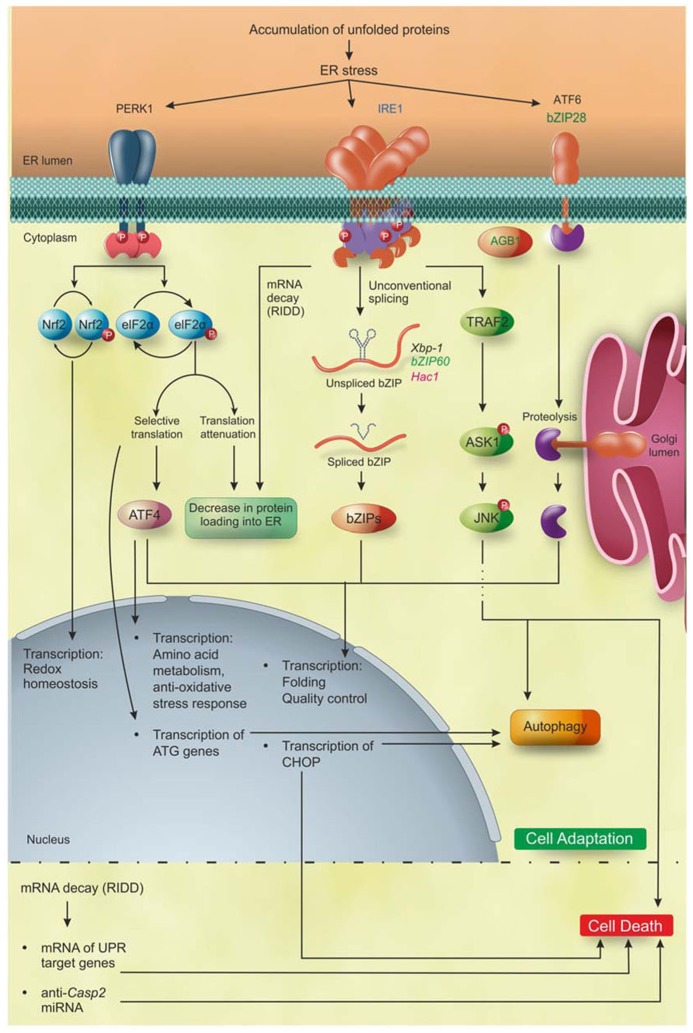
**PR pathways in eukaryotes.** Accumulation of unfolded proteins inside the ER lumen triggers to UPR that, during the first layer of response, leads the cell to adaptation. IRE1 is the only identified ER stress sensor in yeast and it is widely conserved in metazoan and plants. Moreover, additional ER stress sensors so far identified are bZIP28 and AGB1 in plants, and ATF6 and PERK1 in metazoan. Activation of PERK leads to phosphorylation of two proteins: Nrf2 that activates the transcription of genes involved in redox homeostasis; and eIF2α that decreases the overload of newly synthetized proteins into the ER and increases the specific translation of the transcription factor ATF4. Subsequently, ATF4 induces the expression of genes involved in amino acid metabolism, anti-oxidative stress response, folding, quality control and autophagy. The activation of IRE1 leads (1) to the selective degradation of mRNAs (RIDD) encoding ER-translocating proteins, decreasing the protein loading into the ER, and (2) to the translation of the bZIP transcription factors Xbp1/bZIP60/Hac1 mRNA in metazoan, plants and yeast, respectively, that upregulate the UPR target genes mainly involved in folding, quality control and autophagy. ATF6 in metazoans and bZIP28 in *Arabidopsis* are translocated into the Golgi apparatus and cleaved by Site1 and Site2 proteases, releasing the ATF6/bZIP28 transcription factors domain, which then translocates to the nucleus where it increases the expression of genes involved in folding, quality control and autophagy. Upon excessive and prolonged ER stress, all the UPR pathways lead to cell death in yeast, metazoan, and plants. In plants, the cell death executioners are still largely unknown, while in metazoan they have been partially defined. In detail, IRE1 regulates the cell death through the mRNA decay of UPR target genes and of anti-apoptotic protein (anti-Casp2). In addition, IRE1 interacting with TRAF2 triggers the ASK1/JNK pathway promoting apoptosis. Moreover, PERK/eIF2α/ATF4, IRE1, and ATF6 induce the expression of the transcription factor CHOP, involved in the induction of apoptosis. Blue, eukaryotes; black, metazoan; green, plants; pink, yeast. Figure adapted from Chen and Brandizzi (2012).

The different arms of the UPR have evolved to activate overlapping but non-identical pathways in order to restore homeostasis, or, if the ER stress persists, to trigger programed cell death (PCD) in plants and yeasts, and apoptosis in metazoans (**Figure 2**). In the early phases of ER stress responses in metazoans, the overload of newly synthesized proteins into the protein-overloaded ER is reduced through the selective degradation of many mRNAs encoding ER-translocating proteins by the IRE1 endonuclease activity, a process termed “regulated IRE1 dependent decay” (RIDD), and through the transient attenuation of global protein translation via PERK. RIDD promotes the rapid mRNA decay of genes encoding secretory proteins (Hollien and Weissman, 2006; Han et al., 2009) and a similar mechanism most likely operates in plants (Mishiba et al., 2013). Interestingly, RIDD-mediated decrease in ER protein overload was also demonstrated in fission yeast *Schizosaccharomyces pombe*, where it functions as the exclusive UPR mechanism (Kimmig et al., 2012), but not in the budding yeast *Saccharomyces cerevisiae*, where, indeed, the UPR does not appear to attenuate protein translation (Spear and Ng, 2003).

To attenuate ER stress in metazoans in addition to RIDD, PERK phosphorylates the α subunit of the eukaryotic translation initiation factor 2 (eIF2α), inhibiting the 80s ribosome assembly and down-regulating protein synthesis (Harding et al., 1999). In mammals, the phosphorylation of eIF2α is a conserved mechanism to block general protein translation not solely restricted to ER stress, and it is carried out by different kinases activated by diverse cellular stresses. The only eIF2 α-kinase conserved among eukaryotes is the GCN2 protein: GCN2/eIF2 α pathway attenuates protein translation under nutrient limitation in yeasts and mammals (Berlanga et al., 1999; Harding et al., 2000), and under amino acid starvation, abiotic and biotic stresses and plants (Lageix et al., 2008; Zhang et al., 2008). Whether the plant GCN2 may function as the metazoan PERK in the UPR is yet unknown.

## THE ADAPTIVE CELLULAR RESPONSE

Once the first layer of response to ER stress conditions is completed, in plants, metazoans and yeasts all the UPR stress sensors promote a coordinated adaptive response to protect the cell against oxidative stress, to augment protein-folding and secretory capacity in order to ensure that protein exit the ER productively, and to degrade potentially toxic unfolded proteins, by up-regulating the genes encoding oxido-reductases, ER chaperones, vesicle trafficking proteins, ER-associated degradation (ERAD) and ER-quality control (ERQC) components (Travers et al., 2000; Martínez and Chrispeels, 2003; Kamauchi et al., 2005).

### IRE1 AND ITS UNCONVENTIONAL RNA-SPLICING ARM

The IRE1 endonuclease domain catalyzes the non-conventional cytoplasmic splicing of the mRNA encoding *bZIP60/XBP1/HAC1* (in *Arabidopsis*, metazoans and budding yeast respectively), leading to the translation of a transcription factor (bZIP60s/XBP1s/HAC1s) that mainly upregulates the expression of ERQC and ERAD-related genes (Yoshida et al., 2001; Iwata et al., 2008; Deng et al., 2011). Among the species, the amino acid sequences of these transcription factors are not highly conserved; however, a two stem–loop structure accompanied by a consensus sequence in each loop of the IRE1-splicing mRNA substrates is remarkably conserved and associated with IRE1-mediated cleavage (Oikawa et al., 2010; Deng et al., 2011; Nagashima et al., 2011). In yeast and metazoans, the spliced substrate becomes a potent transcriptional activator, since it gains a transcriptional activation domain in the new C-terminal tail (Mori et al., 2000; Yoshida et al., 2001). It is noteworthy that in the absence of induced ER stress in budding yeasts, the unspliced *HAC1* (*HAC1u*) mRNA is not translated, since an intron in the *HAC1u* mRNA blocks its translation (Chapman and Walter, 1997), while in mammalian cells the unspliced *XBP1* (*XBP1u*) mRNA is translated. XBP1u protein along with the *XBP1u* mRNA is associated peripherally with the ER membrane through an amphipathic region, where it facilitates the targeting of *XBP1u *mRNA to IRE1 presumably to increase the cytoplasmic splicing efficiency providing a rapid response to ER stress (Yanagitani et al., 2009). Upon prolonged ER stress, XBP1u forms a complex with XBP1s, leading it to be exported from the nucleus to the cytoplasm and rapidly degraded by the proteasome, presumably shutting down the transcription of the XBP1-target genes (Yoshida et al., 2006). Intriguingly, the IRE1-spliced *XBP1s *mRNA loses the ER membrane-anchor domain and it is released in the cytosol, indicating a different translational place for *XBP1u* and *XBP1s* mRNAs that presumably prevents the excess of degradation of the XBP1s by XBP1u protein during ER stress (Yanagitani et al., 2009). In plants, experiments based on the expression of in-frames fluorescent protein fusion with unspliced bZIP60u, have shown an association of bZIP60u with the ER through its putative C-terminal transmembrane domain (Deng et al., 2011). However, the biological roles of unspliced bZIP60 (bZIP60u) on the ER membrane are currently unknown. Unlike yeast and metazoans, spliced bZIP60 (bZIP60s) does not gain a transcriptional activation activity, since the transcriptional activation domain is located in the N-terminal tail along with a nuclear localization signal. The IRE1-splicing produces instead a new protein deprived of the transmembrane domain (Deng et al., 2011; Nagashima et al., 2011) and characterized by an improved fine regulatory modulation of its transcription activity as recently reported in rice (Lu et al., 2012). However, how this tuning is achieved is still not clear.

### ATF6-LIKE TRANSCRIPTION FACTORS

Under ER stress, the transcription factor domain of bZIP28/ATF6 increases the expression of genes involved in protein folding and of other ER-stress related transcription factors such as bZIP60/XBP-1, providing a positive feedback for augmenting the UPR (Yoshida et al., 2001; Liu and Howell, 2010). Moreover, in metazoans, ATF6 enhances the expression of genes involved in ERAD, lipid biosynthesis and ER expansion, which are required to improve the capacity of the secretory pathway (Bommiasamy et al., 2009).

### CONSERVED *cis*-ELEMENT IN THE UPR TARGET GENES

The UPR genes are induced through the recognition of *cis*-acting elements on their promoter regions by the UPR transcription factors. In budding yeast, HAC1s binds and activates the UPR element-I (UPRE-I, consensus region CAGNGTG; Mori et al., 1996) and UPRE-II (TACGTG; Fordyce et al., 2012). In mammalians, XBP1 efficiently binds the UPRE-I (GA-TGACGT-G[G/A]; Wang et al., 2000; Yamamoto et al., 2004) and the ER stress responsive element-II (ERSE-II, ATTGG–N–CCACG; Kokame et al., 2001; Yamamoto et al., 2004), while ATF6 binds the ERSE-I (ERSE-I, CCAAT–N9–CCACG; Roy and Lee, 1999) and the ERSE-II (ATTGG–N–CCACG; Kokame et al., 2001) only in the presence of the nuclear transcription factor NF-Y (Yamamoto et al., 2004). Interestingly, the mammalian ERSE-I and UPRE-I elements are conserved in plants (Oh et al., 2003; Iwata et al., 2008; Liu and Howell, 2010), where other *cis*-elements have also been found, such as the pUPRE-II (GATGACGCGTAC; Hayashi et al., 2013) and the pUPRE-III (TCATCG; Sun et al., 2013). Similar to ATF6 in mammals, bZIP28 binds the ERSE-I element with assistance from the transcription factors NF-Y (Liu and Howell, 2010), while bZIP60s directly binds the pUPRE-III (Sun et al., 2013) and it regulates also promoters containing the *cis*-element ERSE-I and UPRE-I (Iwata and Koizumi, 2005). The multiple *cis*-elements involved in the ER stress response and their differential binding affinities for the UPR transcription factors presumably fine temporal modulate the UPR signaling and the ER stress response. Moreover, plants have evolved an additional layer of UPR regulation. The plant-specific nuclear transcription factor *NAC103* is indeed induced by ER stress presumably through bZIP60s, and the encoded protein NAC103 in turn regulates the UPR downstream genes (Sun et al., 2013). However, the precise mechanisms of the gene regulation networks are largely unknown.

### PERK AS AN OXIDATIVE STRESS-ATTENUATOR

In metazoans, although PERK induces general protein translation attenuation, it also favors selective protein translation. Specifically, the PERK-phosphorylated eIF2α activates the translation of mRNAs with uORF (upstream open reading frame) within their 5’ untranslated region (UTR), like the activating transcription factor 4 (ATF4; Harding et al., 2000). ATF4 protects cells against oxidative stress and ensures the supply of reducing substances (i.e., glutathione) by enhancing the metabolism of their precursors (i.e., sulfur-containing amino acids; Harding et al., 2003). Also PERK phosphorylates the transcription factor Nrf2 (nuclear factor erythroid2-releated factor 2), which translocates to the nucleus, heterodimerizes with the small Maf proteins and activates the transcription of genes involved in the redox homeostasis by binding to the antioxidant response elements on the target gene promoters (Cullinan et al., 2003).

### AUTOPHAGY AS A PRO-SURVIVAL MECHANISM

Autophagy is an evolutionarily conserved process of bulk degradation, whereby large portions of cytoplasmic and organellar components are engulfed by double membrane vesicles (termed as “autophagosome”) and delivered to the lysosome in metazoan, or to the vacuole in yeast and plants, for degradation and recycling of macromolecules (Liu and Bassham, 2012). During ER stress, autophagy is activated in yeast, mammals and plants, and it is involved in clearing unfolded protein from the ER by supplementing the ERAD pathway and, in turn, alleviating stress (Ding et al., 2007; Liu et al., 2012). In yeast, IRE1 regulates autophagy through the splicing of *HAC1*, which induces the production of Atg8p, an ubiquitin-like protein required for autophagosome formation (Yorimitsu et al., 2006). Unlike yeasts, in metazoans, autophagy is triggered by the kinase activity of IRE1 and PERK. IRE1, indeed, recruits the adaptor protein TNFR-associated factor2 (TRAF2) on the ER membranes, thus triggering the activation of the apoptosis signal regulating kinase1 (ASK1), which in turn regulates the activation of the c-Jun-N-terminal kinase (JNK), whose pathway induces the autophagosome formation (Pattingre et al., 2009). Moreover, PERK promotes autophagy through the phosphorylation of eIF2α, which induces the expression of ATF4, recently considered a key signal for autophagy activation (Matsumoto et al., 2013). ATF4 in turn activates the expression of autophagy-related (ATG) genes and of C/EBP-homologous protein (CHOP) transcription factor. CHOP and ATF4 together promote and modulate the induction of genes implicated in the formation, elongation and function of the autophagosome (B’chir et al., 2013). Similar to metazoans, in plants, it has been recently found that IRE1, specifically the IRE1b isoform, activates autophagy upon ER stress response independently from the IRE1-mediated *bZIP60* mRNA splicing (Liu et al., 2012). However, the mechanistic features of plant autophagy under ER stress are mainly unknown, in terms of the upstream regulator/s of IRE1b as well as its downstream targets.

## UNRESOLVED ER STRESS LEADS TO CELL DEATH

### UPR AND APOPTOSIS IN METAZOAN

Upon excessive and prolonged ER stress, in metazoans, all the three UPR signaling pathways lead to cell death through apoptosis via the intrinsic mitochondrial pathway. Indeed, UPR regulates the activity of the pro-apoptotic members of the Bcl-2 family via transcriptional and post-transcriptional mechanisms, leading the BAX/BAK-mediated pore formation in the mitochondrial outer membrane, release of cytochrome c from the mitochondria, and subsequent activation of caspases, which are critical regulators of apoptosis via their role in propagating apoptotic signaling cascades (Rodriguez et al., 2011). However, it is not yet clear whether other types of cell death occur to eliminate terminally compromised cells under irreversible ER stress. Intriguingly in metazoans, under prolonged ER stress autophagy may switch from a pro-survival process to apoptosis. Several regulators of autophagy machinery are indeed involved in the apoptosis, such as JNK and CHOP (Rodriguez et al., 2011).

### RIDD AS A PRO-APOPTOTIC EXECUTIONER

In mammals RIDD activity mediated by IRE1α enhances (1) ER stress intensity through the decay of mRNA encoding UPR target genes during the transition phase between the adaptive and apoptotic response (Han et al., 2009), and (2) expression of pro-apoptotic proteases, like the Caspase 2 (Upton et al., 2008), through the decay of selected antiapoptotic pre-miRNAs during the apoptotic response (Upton et al., 2012). In plants, the biological significance of RIDD activity in cell fate determination is still unknown.

### ER STRESS INDUCED-CELL DEATH IN YEASTS AND PLANTS

In yeast, ER stress can induce PCD with apoptotic phenotypes (Hauptmann et al., 2006), as well as in a non-apoptotic process, where vacuole fragmentation and leaking of vacuolar materials are cell death features (Kim et al., 2012). Plant cell death executioners in the UPR are instead largely unknown. Unlike in metazoans, the plant IRE1 does not seem to have a pro-apoptotic role, given that the *Arabidopsis ire1* double mutants display compromised ER stress tolerance, instead of a greater survival rate (Nagashima et al., 2011; Chen and Brandizzi, 2012). Although neither homologs of Bcl-2 family proteins nor components of the PERK-CHOP pathways have been identified in plants yet, some regulators of ER-PCD seem to be conserved across kingdoms. These include the Bax inhibitor1 (BI-1)-like protein, an ER transmembrane protein that protects cells against ER-stress induced-cell death (Chae et al., 2003; Watanabe and Lam, 2008), and the chaperone BiP, that has a protective function against ER stress induced-cell death in both mammalian and plant cells (Kishi et al., 2010; Reis et al., 2011).

## OTHER FACTORS THAT CONTROL THE UPR IN PLANTS

Plants have developed UPR roles for evolutionarily conserved gene family, like for the Bcl-2-associated athanogene7 (BAG7) protein, and AGB1, the Gβ subunit of heterotrimeric GTP-binding protein family. In detail, BAG7 a plant ER-localized protein involved in the UPR aids chaperones like BiP in the protection of cells via a co-chaperone activity, while in yeast and mammals BAGs have a nuclear/cytoplasmic localization and are not involved in the maintenance of the UPR, but rather in other processes ranging from proliferation to growth arrest and cell death (Williams et al., 2010). Furthermore, in plants, AGB1 and IRE1 have antagonistic roles in the UPR gene induction and they regulate essential and independent UPR signaling arms (Wang et al., 2007; Chen and Brandizzi, 2012, 2013b), but the underlying mechanisms are unclear.

## CONCLUDING REMARKS

Environmental conditions, such as heat and salt stresses (Liu et al., 2007b; Deng et al., 2011), as well as physiological events, like growth and developmental processes (Chen and Brandizzi, 2012; Deng et al., 2013) evoked ER stress. Moreover, UPR has been recently linked to the phytohormone auxin, a master regulator of plant physiology, revealing a plant-specific strategy to maintain balance between stress adaptation and growth regulation (Chen et al., 2014). Additional studies are required to elucidate the plant UPR signaling and its molecular components, and how it is fine regulated during physiological events and environmental stresses. Also further work is needed to clarify the mechanisms leading the UPR to switch from cell survival to cell death and to identify the precise steps downstream of each UPR arm across different kingdoms.

## Conflict of Interest Statement

The authors declare that the research was conducted in the absence of any commercial or financial relationships that could be construed as a potential conflict of interest.
